# Pragmatic cluster randomised controlled trial of facilitated family case conferencing compared with usual care for improving end of life care and outcomes in nursing home residents with advanced dementia and their families: the IDEAL study protocol

**DOI:** 10.1186/s12904-015-0061-8

**Published:** 2015-11-21

**Authors:** Meera Agar, Elizabeth Beattie, Tim Luckett, Jane Phillips, Georgina Luscombe, Stephen Goodall, Geoffrey Mitchell, Dimity Pond, Patricia M. Davidson, Lynnette Chenoweth

**Affiliations:** Discipline of Palliative and Supportive Services, Flinders University, Adelaide, Australia; South West Sydney Clinical School, and Improving Palliative Care through Clinical trials (ImPACCT), University of New South Wales, Sydney, Australia; Department of Palliative Care, Braeside Hospital, HammondCare, Sydney, Australia; Ingham Institute of Applied Medical Research, Sydney, Australia; Dementia Collaborative Research Centre, Queensland University of Technology, Brisbane, Australia; School of Nursing, Midwifery and Social Work, University of Queensland, Brisbane, Australia; Centre for Cardiovascular and Chronic Care, Faculty of Health, University of Technology Sydney, Sydney, Australia; School of Rural Health, Faculty of Medicine, The University of Sydney, Sydney, Australia; Centre for health Economics Research and Evaluation (CHERE), University of Technology Sydney, Sydney, Australia; Faculty of Medicine and Biomedical Sciences, University of Queensland, Brisbane, Australia; School of Medicine and Public Health, Faculty of Health, University of Newcastle, Newcastle, Australia; School of Nursing, Johns Hopkins University, Baltimore, USA; Faculty of Health, University of Technology Sydney, Sydney, Australia; Centre for Healthy Brain Ageing, University of New South Wales, Sydney, Australia; University of Technology Sydney (UTS) Faculty of Health, Building 10, Level 7, 235-253 Jones St, Ultimo, NSW 2007 Australia

**Keywords:** End of life care, Palliative care, Dementia, Nursing homes, Person-centred care, Case conferencing, Advance care planning, Communication, Implementation, Cost effectiveness

## Abstract

**Background:**

Care for people with advanced dementia requires a palliative approach targeted to the illness trajectory and tailored to individual needs. However, care in nursing homes is often compromised by poor communication and limited staff expertise. This paper reports the protocol for the IDEAL Project, which aims to: 1) compare the efficacy of a facilitated approach to family case conferencing with usual care; 2) provide insights into nursing home- and staff-related processes influencing the implementation and sustainability of case conferencing; and 3) evaluate cost-effectiveness.

**Design/Methods:**

A pragmatic parallel cluster randomised controlled trial design will be used. Twenty Australian nursing homes will be randomised to receive either facilitated family case conferencing or usual care.

In the intervention arm, we will train registered nurses at each nursing home to work as Palliative Care Planning Coordinators (PCPCs) 16 h per week over 18 months. The PCPCs’ role will be to: 1) use evidence-based ‘triggers’ to identify optimal time-points for case conferencing; 2) organise, facilitate and document case conferences with optimal involvement from family, multi-disciplinary nursing home staff and community health professionals; 3) develop and oversee implementation of palliative care plans; and 4) train other staff in person-centred palliative care.

The primary endpoint will be symptom management, comfort and satisfaction with care at the end of life as rated by bereaved family members on the End of Life in Dementia (EOLD) Scales. Secondary outcomes will include resident quality of life (Quality of Life in Late-stage Dementia [QUALID]), whether a palliative approach is taken (e.g. hospitalisations, non-palliative medical treatments), staff attitudes and knowledge (Palliative Care for Advanced Dementia [qPAD]), and cost effectiveness. Processes and factors influencing implementation, outcomes and sustainability will be explored statistically via analysis of intervention ‘dose’ and qualitatively via semi-structured interviews. The pragmatic design and complex nature of the intervention will limit blinding and internal validity but support external validity.

**Discussion:**

The IDEAL Project will make an important contribution to the evidence base for dementia-specific case conferencing in nursing homes by considering processes and contextual factors as well as overall efficacy. Its strengths and weaknesses will both lie in its pragmatic design.

**Trial registration:**

Australian New Zealand Clinical Trials Registry (ANZCTR) ACTRN12612001164886. Registered 02/11/2012.

## Background

Dementia is a chronic, progressive and terminal illness that requires a palliative approach to care in its advanced stages [1–3]. Maximising quality of life (QOL) for persons with advanced dementia and their families requires careful and regular assessment of physical, psychosocial, cultural and spiritual needs at timely junctures in the illness trajectory to inform management decision-making and advance care planning. Symptom assessment and management must be tailored to the specific needs of advanced dementia, with an understanding that deterioration is often complicated by comorbid conditions. Pneumonia and feeding problems are common and require focused management and planning. People with advanced dementia have limited capacity to effectively communicate their needs and participate in decisions about care, such that symptoms (e.g. pain) often go under-recognised and under-managed [4–6]. Interventions for acute medical problems (e.g. intravenous antibiotics/hydration, tube feeding) should be applied only at times where they will provide net benefit, rather than compromise comfort and distress families for little or no survival benefit [7–13].

Delivery of palliative care to nursing home residents with advanced dementia is often suboptimal, either because staff lack awareness that a palliative approach is required or else find it difficult to apply this to dementia care [2, 4–8, 12, 14–17]. Major barriers include an inadequate skill-mix, poor communication between nursing home staff, health services and families, and inadequate planning and/or inconsistency in decision-making [4, 15]. If an advance care plan is not in place, staff may be unable or unwilling to manage symptoms within the nursing home, leading to unnecessary transfer to hospital [18]. Hospital admissions for people with dementia are more likely to be lengthy and terminal compared to those for people without [19]. Hospitalisation is often frightening for residents and is associated with care focused on acute medical conditions rather than QOL [20, 21]. Acute care staff may have limited knowledge about dementia and the person’s unique needs, and family may have a reduced role in decision-making [22].

Case conferencing has been promoted as a promising approach for improving care outcomes for people with dementia living in nursing homes [23, 24]. It brings together relevant health professionals and other decision-makers to discuss healthcare problems with the person and their family, provides a formal framework to understand expected changes, and can build consensus on goals of care for advance care planning. For residents with advanced dementia who cannot speak for themselves, case conferences can facilitate the sharing of different perspectives regarding what each resident would have wanted were (s)he able to choose. The lack of resident capacity for decision-making mandates involvement of family members with legal authority to make decisions on behalf of the resident. Moreover, involvement of family in decision-making may lead to improvements in their satisfaction with care in and of its own right [12]. This fits well with the philosophy of a palliative approach more generally, which assumes that the unit of care includes family members as well as the person with life-limiting illness him/herself. However, direct evidence for a relationship between case conferencing and improved QOL in nursing home residents with dementia is limited to a cross-sectional survey study [25]. While two recent systematic reviews [23, 24] identified five randomised controlled trials (RCTs) of case conferencing in nursing homes [26–30], none were concerned with palliative care or care for residents with advanced dementia. Moreover, the case conferencing approaches used in these RCTs did not include family decision-makers, and only two included health professionals from disciplines other than nursing [26, 27].

Stronger evidence for an impact from case conferencing on palliative care quality and outcomes is available in the community palliative care setting, where two RCTs have found case conferencing to lead to better maintenance of physical and mental health [31] and decrease hospitalisations [32]. The interventions in these studies facilitated case conferencing via strategies for building staff knowledge and motivation to undertake case conferencing, providing staff with detailed guidance for care planning, obtaining organisational and administrative support for case conferencing, and facilitating general practitioner attendance [33–35]. The positive results from these trials highlight the structure and support needed for case conferencing to succeed in the community setting. Support of this kind may be even more important in the nursing home setting where staff are sometimes confused about the purpose of case conferencing and its role in healthcare, uncertain about the need for multidisciplinary input and the role of different disciplines, and where there is often a lack of collaborative culture [36, 37].

### Aims

The current paper reports the protocol for a cluster RCT called the IDEAL Project (Implementing Dementia End of life care At Local aged care facilities). This project has been designed to: 1) compare the efficacy of a facilitated approach to family case conferencing with usual care with regard to end of life (EOL) care and outcomes for residents with advanced dementia living in nursing homes; 2) provide insights into nursing home- and staff-related processes influencing the implementation and sustainability of case conferencing; and 3) evaluate the cost-effectiveness of facilitated family case conferencing versus usual care.

### Hypotheses

#### Primary hypothesis (Aim 1)

Compared with usual care, facilitated family case conferencing for residents with advanced dementia will achieve better family-rated EOL outcomes as defined by: a) better symptom-related comfort in the last 7 days of life; b) more effective symptom management over the last 90 days of life; c) greater family satisfaction with care over the last 90 days of the resident’s life.

#### Hypothesis 2 (Aim 2)

Compared with usual care, facilitated family case conferencing for residents with advanced dementia will achieve:

- improved nurse-rated symptom-related comfort over the last 7 days of life and symptom management over the last 90 days of life;
- higher scores on resident QOL at last measurement prior to resident death;
- a palliative approach to medical care of residents with advanced dementia, as evidenced by reduced acute care episodes, non-palliative interventions and adverse incidents at the nursing home level;
- a person-centred approach by nursing home staff to all aspects of care;
- improvements in nursing home staff attitudes to, knowledge of and confidence in providing palliative care to residents with advanced dementia and dementia care more generally.
- improvements in family QOL.

#### Hypothesis 3 (Aim 3)

Compared with usual care, facilitated family case conferencing for residents with advanced dementia will result in EITHER an overall reduction in health care costs with no loss to resident health OR be cost-effective in terms of health gained at acceptable additional cost.

## Methods

### Development of the intervention

The IDEAL Project was informed by the Medical Research Council’s (MRC) framework for complex interventions [38]. In a *development phase*, a case conferencing toolkit was compiled by members of the team for use in a general nursing home population [39]. During a *feasibility and piloting ph*ase, the toolkit and method of implementation was subjected to preliminary testing for residents with advanced dementia in six nursing homes in metropolitan and rural New South Wales [40]. The pilot demonstrated the acceptability and clinical relevance of the toolkit, dementia-specific “triggers” for conduct of case conferences based on resident characteristics and/or clinical events (e.g. on admission to the nursing home, change in clinical status), and a palliative care framework for mapping the trajectory of residents in specific domains of disease progression (functioning, swallowing, weight loss, continence, level of consciousness, communication, and factors related to comorbid illness) and identifying issues that case conferencing should address. The pilot also informed development of a list of competencies for the role of case conference coordinator and associated training materials for the nursing home staff, and demonstrated feasibility of collecting descriptive data and outcome measures in the *evaluation phase*. Baseline data collected in the pilot also confirmed previous findings that, without facilitation, case conferences may seldom occur and/or be of poor quality [36, 37].

### Study design

The IDEAL Project will use a parallel cluster RCT design because the intervention requires system-level changes that make it impossible to randomise individual residents within the same nursing home to different arms without contamination [41].

We follow previous trials of case conferencing [42] in taking a pragmatic approach to design with the aim of improving external validity [43, 44]. Features of pragmatic trials considered valuable by the project team include: 1) broad inclusion criteria to reflect the diversity of nursing homes and residents with advanced dementia and multiple comorbidities; 2) tailoring of the intervention to local conditions and individual resident needs; and 3) selection of endpoints focused on clinically important outcomes. We suggest that these benefits outweigh the disadvantages of a pragmatic design, namely poorer internal validity and inefficiency, as discussed below.

Ethics approval has been granted by the Human Research Ethics Committee (HREC) of the University of New South Wales and ratified by HRECs at the University of Technology Sydney and Queensland University of Technology.

### Randomisation and blinding

Block randomisation of nursing homes will use a computer generated allocation sequence and occur after initial consent. Randomisation will be stratified by organisational affiliation to control for the influence of organisational culture, policy and procedures on quality of care. Randomisation will also be stratified by rurality and dementia-specificity following evidence that these variables may be associated with quality of care for residents with advanced dementia [18].

The pragmatic nature of the trial and complex nature of the intervention requires careful consideration of the feasibility versus desirability of blinding [43, 44]. The statistician (GL) will be blinded to nursing home identity for randomisation and analysis. However, nursing home managers will not be blinded to allocation because they need to make a fully informed decision regarding nursing home participation. Nursing staff and families at nursing homes in the intervention arm will also know that facilitated case conferencing is being implemented because they will be participating. The design seeks to limit potential for bias by omitting from participant information any reference to the study’s evaluative aims or allocation of nursing homes to intervention versus usual care arms. Instead, verbal and written information will describe the study’s aim in more general terms as being concerned with understanding the factors associated with quality of care for residents with advanced dementia by comparing between nursing homes with different characteristics. Research assistants collecting process and outcome data will also be blinded to the specific design and aims of the study, and each assistant will visit nursing homes in either the intervention or usual care arm only. However, the lead investigator (MA), the national project coordinator (TL), and two state-based project managers will remain unblinded for the purpose of liaising with implementation personnel and ensuring protocol fidelity.

### Sites

The study will be conducted in nursing homes in two Australian capital cities (Sydney, New South Wales, and Brisbane, Queensland). Nursing home inclusion criteria are designed to ensure sufficient numbers of eligible residents and are: 1) ≥100 beds, 2) ≥50 % residents with dementia, and 3) designation as ‘high care’ (i.e. the most intensive level of care provided by Australian nursing homes). To minimise selection bias, nursing homes will be identified from listings on an Australian government website [45] and approached in random order.

### Participants and recruitment

Residents will be eligible to participate if they have a diagnosis of dementia documented in nursing home records and identified as having advanced disease as determined by: 1) a score on the Functional Assessment Staging Tool (FAST) [46] in dementia of ≥6a that has been stable for 1 month, and 2) a score on the Australia–modified Karnofsky Performance Status (AKPS) [47] of ≤50 (i.e. a performance status of ‘considerable assistance and frequent medical care required’ or worse). The FAST was chosen because of its utility in the advanced stages of dementia and its prognostic value; a FAST stage 7c combined with functional dependency (measured here by the AKPS) is predictive of an average survival of <6 months [48]. Potentially eligible residents will be identified by nursing home staff and screened by the project team.

Because residents will lack capacity to provide informed consent, this will be sought on their behalf from someone with designated authority as defined by legislation in New South Wales (‘person responsible’) and Queensland (‘statutory health attorney’). For each participating resident, written informed consent will be obtained additionally from a family member who visits the resident regularly and has authority to be involved in decisions about the resident’s care. This person can be the same or different to the person responsible/statutory health attorney who gives consent for the resident to participate.

Permanent nurses and care assistants who provide care for enrolled nursing home residents will be invited to participate via group emails and face-to-face staff meetings. Community health professionals (e.g. GPs, community nurses, allied health professionals) attending case conferences for participating residents will be invited to provide written informed consent to participate in exit interviews and to allow case conferences to be audio-recorded for content analysis.

### Conditions

The intervention and usual care arms to be compared in this study are as follows.

#### Intervention – facilitated case conferencing

The intervention utilises elements of two models for bringing about change in organisational culture, namely clinical leadership [49] and train-the-trainer [50]. A Palliative Care Planning Coordinator (PCPC) will be appointed at each nursing home and trained to work on the project in a funded capacity for 16 h (0.4 full time equivalent) per week. Because change may be more sustainable when led from within an organisation, PCPCs will be appointed from existing nursing staff at intervention nursing homes based on criteria relating to clinical training and expertise (typically a senior registered nurse), relationships with staff, and identification by managers as a 'change champion' using established criteria [51].

As part of their orientation to the role, PCPCs will attend one week (35 h) of interactive training in which they will be provided with materials and tuition on the principles of person-centred palliative care for people with dementia as well as the organisation, conduct and documentation of case conferences and person-centred care plans. Materials will be based on a previous resource developed by members of the team for case conferencing in aged care more generally [39], recently adapted to meet the specific needs of residents with advanced dementia and their families. In practical terms, PCPCs will be trained to: 1) use evidence-based ‘triggers’ to identify residents with advanced dementia at a time-point likely to benefit from a case conference (Table 1); 2) organise, set an agenda, facilitate and document case conferences with optimal involvement from family, multi-disciplinary nursing home staff and external health professionals (e.g. GPs); 3) develop and oversee implementation of palliative care plans; and 4) train other nursing home staff in person-centred palliative care. Consistent with MRC guidance for complex interventions [38], training will focus on how the intervention might best be adapted to local conditions at each nursing home and integrated within existing initiatives. Training will be run by a multidisciplinary team including physicians, nurses and consumers. It will make use of experiential learning as well as didactic approaches, and illustrate key learning objectives with case studies.

**Table 1:** Triggers prompting the organisation and conduct of a case conference

• Admission to the nursing home	
• Return to the nursing home following discharge from acute hospital	
• Increase in falls	
• Change in clinical status	
• New/worsening symptoms	
• Poor appetite or skin integrity	
• Annual management plan review	
• Receipt of a complaint	
• Family disagreement about care	
• Family distress	

Following training, PCPCs will be supported on an ongoing basis by means of bi-weekly teleconferences aimed at building peer support and group learning via a community of practice [52], as well as individual telephone support and site visits from the project team as required.

#### Usual care

In nursing homes, randomised to the usual care arm, no education, training or support will be provided by the study team, but neither will any restriction be placed on service provider education or training where this is current practice. The level, duration and type of service provider initiatives will be documented so these can be controlled for as potential confounders.

### Data collection

#### Baseline data

##### Nursing home, staff, resident and family characteristics

Characteristics that have potential to influence outcomes will be collected as per Table 2 via nursing home records and staff/family report.

**Table 2:** Data collected on characteristics of facilities, residents, families and staff with potential to influence outcomes

Level	Time point(s)	Variables/measures
Facility	Baseline	Number of beds; high care versus low care; private versus not-for-profit status; organisational affiliation; dementia-specific status; accommodation and amenities; management structure; person-centredness (PCECAT)
	Baseline and 3 monthly	Number of new admissions and deaths; number and proportion of residents with dementia and advanced dementia; proportion of residents requiring complex care
		Involvement of GPs and other community services; rates of acute care episodes and length of stay (including ED presentations with admission and actual admission); rates of potentially non-palliative interventions* and intra-venous anti-biotics
		Staff profile and turnover (resignations and new staff); resident to staff ratio; use of agency staff; staff training
		Accidents/incidents
Resident	Baseline	Age; gender; previous occupation; years of education
		Time since dementia diagnosis; BMI
		Length of stay in NH
	Baseline and 3 monthly	Dementia stage (FAST); performance statues (AKPS); support needed for activities of daily living (BANS); comorbidities and level of care needs (ACFI); food and fluid intake; palliative care phase (PCOC phase)
		Number of visitors and frequency of visits
	Whole time on study	ED visits and hospitalisations (reason, whether a GP was consulted in decision to hospitalise, length of stay); potentially non-palliative interventions*; goals of care; reports for any RMMRs conducted
	Last month of life	Place of death and(if not NH) reason for transfer and who decided; symptoms; formal symptom assessments; management over last 24 h of life (detailed); non-pharmacological management; input from health professionals; medication changes and rationale
Family	Baseline	Self-reported relationship to resident; age; gender; education; occupation; dependents; frequency/duration of visits; person responsible status** and prior involvement in decision-making
Staff	Baseline	Self-reported age; gender; qualifications; position; time in position; time in NH care; dementia experience and previous staff training
	Baseline and 3 monthly	Knowledge and attitudes towards advanced dementia (qPAD)

ACFI = Aged Care Funding Instrument [78]; AKPS = Australia–modified Karnofsky Performance Status [47]; BANS = Bedford Alzheimer Nursing Severity (BANS) [79]; BMI = body mass index; ED = emergency department; FAST = Functional Assessment Staging of Alzheimer’s Disease [46]; GP = general practitioner; NH = nursing home; PCECAT = Person Centred Environment and Care Assessment Tool [80]; PCOC = Palliative Care Outcomes Collaborative [81]; qPAD = questionnaire on Palliative care for Advanced Dementia [64]; RMMR = residential medication management review; * potentially non-palliative interventions defined as ventilation, resuscitation, nasogastric/ percutaneous endoscopic gastrostomy (PEG) feeding, cardiopulmonary resuscitation, dialysis, oxygen, transfusion; **person responsible status concerns a person’s legal status as a surrogate decision-maker

#### Process and sustainability data

Data will be collected as per Table 3 for use in analyses aimed at measuring ‘dose’ of the intervention and describing facilitative or obstructive mechanisms that may be used to inform future implementation [38]. Complex interventions in nursing homes can be influenced by a large range of variables at the levels of resident, family, staff and nursing home. Capturing information about these variables is important to enable understanding of which groups the intervention may be optimal for, how it might be adapted to meet the needs of other groups, and the broader contexts in which it is likely to be more or less successful.

**Table 3:** Variables collected to measure ‘dose’ of facilitated case conferencing and scoring system used

Facility level dose	Scoring
1. Extent to which PCPC able to work 2 days per week	0 for lesser extent, 1 for moderate extent, 2 for large extent
2. PCPC role diffused through RACF beyond PCPC	0 for lesser extent, 1 for moderate extent, 2 for large extent
3. PCPC reported manager to be supportive	0 for lesser extent, 1 for moderate extent, 2 for large extent
4. Evaluation by project team regarding extent that PCPCs were able to fulfil expectations and roles according to training/handbook	0 for lesser extent, 1 for moderate extent, 2 for large extent
Resident level dose	
1. Number of case conferences	0 for none, 1 for one, 2 for two, 3 for three more
2. Median number of professional carer disciplines other than RN and GP involved	0 for none, 1 for one, 2 for two, 3 for three or more
3. One or more case conference(s) attended by a GP?	0 for no, 1 for yes

#### Outcome data

Intervention and usual care arms will be compared on the following outcomes collected at baseline and specified time-points.

#### Primary endpoint

Family-rated EOL outcomes will be measured using the End of Life in Dementia (EOLD) Scales [53]. These scales have established internal consistency (Cronbach’s alpha = 0.68–0.83) and convergent validity in the nursing home setting [54], and have been rated the best measures for evaluating dementia-specific palliative care in nursing homes [55]. EOLD data from the CASCADE Study (Choices, Attitudes, and Strategies for Care of Advanced Dementia at the End of life) will enable comparison with US residents [56]. The EOLD Scales will be used to measure: a) symptom-related comfort during the last 7 days of life (Comfort Assessment in Dying with Dementia; CAD–EOLD), including physical distress, dying symptoms, emotional distress and wellbeing; b) symptom management in the last 90 days of life (Symptom Management at the End of life in Dementia; SM–EOLD); and c) family satisfaction with care during the last 90 days of life (Satisfaction with Care at the End of life in Dementia; SWC–EOLD). Whilst the CAD-EOLD and SM-EOLD have been validated for rating either by family or nurses, family ratings will be the primary endpoint because family perceptions of EOL suffering and its management are important outcomes for palliative care in and of their own right and family ratings may be less subject to response bias than those of nurses who are delivering care as well as reporting on its quality. All scales will be rated by families 4 to 6 weeks following resident death to avoid rating during the initial period of acute grief [57].

#### Secondary endpoints

- Nurse-rated symptom-related comfort and symptom management will be measured using the CAD-EOLD and SM-EOLD Scales and rated 4 weeks following residents’ death by the nurse who provides most care in the last 7 days and 90 days of life respectively. Nurse proxy ratings will be included because they offer a 24-h observational perspective that complements family proxy ratings [58].
- Proxy nurse-rated resident QOL will be measured second weekly using the Quality of Life in Late-stage Dementia (QUALID) Scale [59]. This 11-item instrument is the only advanced dementia-specific QOL measure and has been specifically designed for proxy rating. It has shown convergent validity, internal consistency (Cronbach’s alpha = 0.77) and test-retest (ICC = 0.81) and inter-rater (k = 0.83) reliability. QUALID data from the Caring for Aged Dementia Care Resident Study (CADRES) [60] and CASCADE [56] will enable comparison with large Australian and US samples. For the purposes of economic evaluation, QOL will also be assessed by proxy using the EQ-5D-5 L [61], a five level version of the world’s most widely used multi-attribute utility instrument EQ-5D [62] which has been revised to include a larger number of severity levels among its response options (therefore giving greater sensitivity). The EQ-5D has been widely used in dementia studies [63].
- A palliative approach to care at the nursing home level will be measured using the following indices: rates of potentially inappropriate non-palliative interventions and acute care episodes and length of stay (including ED presentations with admission and actual admission); rates of inappropriate acute care episodes and inappropriate non-palliative interventions as judged again by an expert review of 10 % of admissions at each nursing home; and number/type of complaints from families regarding the quality of care. Potentially non-palliative interventions will be defined as ventilation, resuscitation, nasogastric/percutaneous endoscopic gastrostomy (PEG) feeding, intravenous antibiotics and fluids, dialysis, transfusion, oxygen and surgery. Adverse events will be defined as falls with/without injury, skin tears, injuries during care, and medication incidents. Source documents will include nursing records, transfer letters, discharge summaries and medical records written on admission and discharge. Where records are not kept, data will be based on report by managers. The 10 % of admissions for expert review will comprise a random sample of admissions over the previous year, focusing on residents with dementia.
- Nursing home staff’s attitudes to, knowledge of and confidence in providing palliative/EOL care to residents with advanced dementia will be evaluated using the questionnaire on Palliative Care for Advanced Dementia (qPAD) [64]. This 35-item scale has demonstrated satisfactory factor structure and internal consistency (Cronbach’s alpha 0.58–0.90). The qPAD will be administered before and after training to PCPCs and at baseline and 3-monthly intervals to participating nursing staff.

#### Endpoints for economic evaluation

The economic evaluation of this trial will take a cost-utility approach, in which health benefit will be estimated in terms of quality adjusted life years (QALYs) gained. This will capture expected intervention effects on morbidity within the context of any unexpected change in survival. Resident QOL will be estimated using nurse-ratings on the EQ-5D-5 L introduced above, for which Australian population preference weights have been valued by a representative sample of the general population [65]. Healthcare costs will be collected from the health care and societal perspectives. Program costs will include training costs (materials, trainer’s time and opportunity cost of trainee time); and case conference costs (session time, travel time and distance travelled by attendees). Health service use during the study period will be extracted from nursing home records, to including medical service utilisation (such as medical practitioner visits and hospital admissions, procedures performed and pharmaceutical drug usage. Total health service costs will be derived by multiplying the units of resource used by the relevant factor: the Australian Government’s Medicare Schedule Benefit item fee, Pharmaceutical Benefits Scheme price, or the Australian Refined Diagnosis-Related Group cost.

#### Qualitative sub-study

A qualitative sub-study will seek to inform in-depth understanding of the processes necessary for implementing case conferencing and the intervention’s acceptability to families, nursing home staff and other participating health professionals. We will also be interested to learn about other factors influencing the quality of EOL dementia care that case conferencing may interact with. Data will be collected via semi-structured interviews conducted at the end of the project with all PCPCs and a random sample of families, nursing home staff and community health professionals caring for enrolled residents in each arm. Semi-structured interviews in both arms will explore perceptions of factors perceived to influence quality of EOL care for residents with advanced dementia. In the intervention arm, interviews will also explore perceptions of the usefulness and facilitators/barriers to implementing case conferencing for people with advanced dementia. PCPCs will also be asked what advice they would give to someone else taking on the role of PCPC and to describe any plans for continuing facilitated case conferencing at their nursing home after the project period. 

### Statistical considerations

#### Sample size

There will be two considerations for sample size in this cluster randomised trial, the number of clusters (nursing homes) and number of participants per cluster. In the absence of established minimal clinically important differences on the EOLD Scales, we follow a common rule-of-thumb for patient reported outcomes and assume 0.5 standard deviation (SD) [66]. Sample size is based on the EOLD scale with the highest intra-cluster correlation (ICC) (i.e., requiring the greatest number of clusters), namely the CAD-EOLD. To detect a change in CAD-EOLD of half a standard deviation (i.e. a change in score of at least 3), with a two-sided 5 % significance level, power of 80 % and an intracluster correlation coefficient of 0.05 (estimated from unpublished data sourced from Dutch nursing homes), a sample size of 8 clusters per group (16 in total) with 15 residents per cluster will be necessary. Given an anticipated dropout rate at the resident level of 10 %, a recruitment sample of 272 residents (17 per site) will be needed for a final sample of 240 residents. We will also over-sample nursing homes by two in each arm (i.e. 10 in each arm, 20 in total) to protect against drop-out by managers. An interim check will be conducted on blinded data at 6 months to assess whether these assumptions require adjustment and whether re-estimation of sample size may be necessary.

The feasibility of enrolling 17 residents with advanced dementia per nursing home with a life expectancy of less than the study period (<18 months) is based on: 1) a review of death data at one large (>200 beds) metropolitan Sydney nursing home, and 2) research showing that 40 % of people admitted to nursing homes with advanced dementia die within 1 year, and 3) resident participants’ eligibility criteria will likely further decrease participant life expectancy [48, 67].

#### Analysis

##### Aim 1 – Efficacy

Primary and secondary analyses will be performed on an intention to treat (ITT) basis. Descriptive statistics will be used to compare characteristics at individual and nursing home-levels in each group at baseline, accounting for the clustering effect in the former.

Mixed or ‘multilevel’ modelling will be used to determine the effects of case conferencing and usual care on primary and individual-level secondary outcomes. Mixed models will allow adjustment for both individual (i.e. resident, family or staff) and cluster-level covariates as well as adjustment for the inherent correlation within clusters.

Cluster level analyses will be implemented to determine the effect of the intervention on nursing home-level outcomes. Analyses will be weighted by cluster size as required. Results will be interpreted and generalised accordingly.

#### Aim 2 – Process and sustainability

Following ITT analyses, further analyses will be conducted that control for the ‘dose’ of intervention received by each nursing home and resident based on variables and scoring outlined in Table 3.

Further understanding of processes and sustainability will be informed by qualitative analysis of interview data, which will use a thematic framework approach [68] that is both deductive (i.e. a ‘top-down’ approach informed by the aims of the research and the structure of the interview topic guide) and inductive (i.e. a ‘bottom up’ approach grounded in the responses of the participants). Analysis will be conducted by two independent researchers using a constant comparative method [69], with any differences in coding resolved by discussion.

#### Aim 3 - Economic evaluation

For the cost-effectiveness component, the benefit of case conferencing relative to usual care will be calculated in terms of the incremental QALYs gained. The cost of the intervention will be measured along with any cost-savings due to avoided healthcare utilisation. Results will be presented in terms of the incremental cost-effectiveness ratio (ICER). Many of these model parameters will not be powered for statistical significance. Therefore, mean estimates of resource utilisation will be used and confidence intervals will be generated by boot-strapping the data. Sensitivity analyses will be undertaken to explore the robustness and validity of the cost-effectiveness data and to test any assumptions used in the economic model.

## Discussion

The cluster randomised trial of palliative care case conferencing described in this article will make an important contribution to the evidence base concerned with family case conferencing for residents with advanced dementia who are dying in nursing homes. As well as being the first to test efficacy and cost-effectiveness, the trial includes measures and analyses of process that will inform understanding of factors influencing implementation of this complex intervention at the levels of nursing home, staff, resident and family.

The study’s design, methods and intervention have been informed by the team’s experience in previous RCTs and other studies of case conferencing and dementia care [31, 34, 35, 70–74]. The study builds on this previous work by: 1) adapting facilitated approaches to case conferencing found efficacious for people with advanced cancer in the community [31, 34, 35]; 2) using evidence-based approaches to teaching person-centred dementia care to nursing home staff [60, 70, 73, 74]; and 3) empowering nursing home staff to bring about change within their own nursing homes by means of clinical leadership, resources and ongoing support [72].

The intervention is aligned with Australian [75] and international [76] policy directions regarding the key elements thought to mediate high quality person-centred dementia and palliative care. It has also been designed specifically to address challenges in the Australian nursing home context relating to variations in workforce profiles, high staff turnover, poor continuity of care, and large numbers of unregulated and untrained personal care workers [77]. It is important that interventions align with business and funding models to maximise potential for sustainable translation into practice and impact on health outcomes that matter to all stakeholders involved. To ensure the study findings are relevant to practice, the resident population will be identified for inclusion using tools currently available for use in clinical practice [46, 47] and associated with the stage of dementia where facilitated case conferencing is hypothesised to have most impact. Where appropriate, opportunities have also been taken to ensure comparability between data collected in this research and the world’s largest study of nursing home care for residents with advanced dementia, the US CASCADE study [56].

The strengths of the IDEAL Project lie in its pragmatic nature [43, 44], which is expected to enhance external validity and inform understanding of effectiveness, implementation, and cost-effectiveness to inform future development and adaptation. On the downside, the proposed design and intervention will enable only partial blinding, and there is likely to be heterogeneity among nursing homes and residents in each arm, limiting internal validity. Collection of detailed information about process is intended to ensure that findings from this research are informative to practice and policy regardless of whether evidence is found for an overall effect on the primary endpoint. As well as disseminating these findings via publications and conference presentations, the researchers expect to produce dementia-specific resources for facilitated case conferencing that will be made available online so they can be kept up to date and accessed freely by the nursing home sector.

### Ethics approval

University of New South Wales Human Research Ethics Committee.

## Abbreviations

AKPS: Australian Karnofsky Performance Status
EOL: End of life
EOLD: End of Life in Dementia
FAST: Functional Assessment Staging Tool
GP: General practitioner
HREC: Human Research Ethics Committee
IDEAL: Implementing Dementia End of life care At Local aged care facilities
PCPC: Palliative Care Planning Coordinator
QALY: Quality adjusted life year
QOL: Quality of life
qPAD: Palliative Care for Advanced Dementia
QUALID: Quality of Life in Late-stage Dementia
RCT: Randomised controlled trial

## References

[1] van der Steen JT, Radbruch L, Hertogh CM, de Boer ME, Hughes JC, Larkin P et al. White paper defining optimal palliative care in older people with dementia: A Delphi study and recommendations from the European Association for Palliative Care. Palliat Med. 2013(Epub ahead of print). 10.1177/0269216313493685 23828874

[2] Mitchell SL, Teno JM, Kiely DK, Shaffer ML, Jones RN, Prigerson HG (2009). The clinical course of advanced dementia. New Engl J Med.

[3] Bosek MS, Lowry E, Lindeman DA, Burck JR, Gwyther LP (2003). Promoting a good death for persons with dementia in nursing facilities: family caregivers' perspectives. JONA'S healthcare law, ethics and regulation..

[4] Chang E, Daly J, Johnson A, Harrison K, Easterbrook S, Bidewell J (2009). Challenges for professional care of advanced dementia. Int J Nurs Pract..

[5] Bayer A (2006). Death with dementia--the need for better care. Age Ageing.

[6] McAuliffe L, Nay R, O'Donnell M, Featherstonehaugh D (2009). Pain assessment in older people with dementia:literature review. J Adv Nurs.

[7] Di Giulio P, Toscani F, Villani D, Bruneeli C, Gentile S, Spadin P (2008). Dying with advanced dementia in long-term care geriatric institutions: a retrospective study. J Palliat Med.

[8] Mitchell SL, Kiely DK, Hamel MB (2004). Dying with advanced dementia in the nursing home. Arch Intern Med.

[9] Sampson EL, Gould V, Lee D, Blanchard MR (2006). Differences in care received by patients with and without dementia who died during acute hospital admission: a retrospective case note study. Age Ageing.

[10] Givens JL, Jones RN, Shaffer ML, Kiely DK, Mitchell SL, Givens JL (2010). Survival and comfort after treatment of pneumonia in advanced dementia. Arch Intern Med.

[11] Meier DE, Ahronheim JC, Morris J, Baskin-Lyons S, Morrison RS (2001). High short-term mortality in hospitalized patients with advanced dementia: lack of benefit of tube feeding. Arch Intern Med.

[12] Engel SE, Kiely DK, Mitchell SL (2006). Satisfaction with End-of-life care for nursing home residents with advanced dementia. J Am Geriatr Soc.

[13] Sorrell JM. Use of feeding tubes in patients with advanced dementia: Are we doing harm? Journal of Psychosocial Nursing and Mental Health Services. 2010;48 (5):15–8. doi:http://dx.doi.org/10.3928/02793695-20100331-02. 10.3928/02793695-20100331-02 20415291

[14] Teno JM (2003). Now is the time to embrace nursing homes as a place of care for dying persons. J Palliat Med.

[15] Birch D, Draper J (2008). A critical literature review exploring the challenges of delivering effective palliative care to older people with dementia. J Clin Nurs.

[16] Hertogh CMPM (2006). Advanced care planning and the relevance of a palliative care approach in dementia. Age and Ageing..

[17] Rurup ML, Onwuteaka-Philipsen BD, Pasman HRW, Ribbe MW, van der Wal G (2006). Attitudes of physicians, nurses and relatives towards end-of-life decisions concerning nursing home patients with dementia. Patient Educ Couns.

[18] Mitchell SL, Teno JM, Intrator O, Feng Z, Mor V (2007). Decisions to forgo hospitalization in advanced dementia: a nationwide study. J Am Geriatr Soc.

[19] Guijarro R, San Roman CM, Gomez-Huelgas R, Villalobos A, Martin M, Guil M (2010). Impact of dementia on hospitalization. Neuroepidemiology.

[20] Hines S, McCrow J, Abbey J, Foottit J, Wilson J, Franklin S (2009). Effectiveness and appropriateness of a palliative approach to care for people with advanced dementia: A systematic review. Dementia Collaborative Research Centre for carers and consumers.

[21] Andrews J, Christie J (2009). Emergency care for people with dementia. Emerg Nurse.

[22] Afzal N, Buhagiar K, Flood J, Cosgrave M (2010). Quality of end-of-life care for dementia patients during acute hospital admission: a retrospective study in Ireland. Gen Hosp Psychiatry.

[23] Reuther S, Dichter MN, Buscher I, Vollmar HC, Holle D, Bartholomeyczik S et al. Case conferences as interventions dealing with the challenging behavior of people with dementia in nursing homes: a systematic review. Int Psychogeriatr. 2012;24(12):1891–903. doi:http://dx.doi.org/10.1017/S1041610212001342. 10.1017/S1041610212001342 22883019

[24] Phillips JL, West PA, Davidson PM, Agar M. Does case conferencing for people with advanced dementia living in nursing homes improve care outcomes: evidence from an integrative review? Int J Nurs Stud. 2013;50(8):1122–35. doi:http://dx.doi.org/10.1016/j.ijnurstu.2012.11.001. 10.1016/j.ijnurstu.2012.11.001 23200128

[25] Beer C, Flicker L, Horner B, Bretland N, Scherer S, Lautenschlager NT et al. Factors associated with self and informant ratings of the quality of life of people with dementia living in care facilities: a cross sectional study. PLoS ONE. 2010;5(12):e15621. doi:http://dx.doi.org/10.1371/journal.pone.0015621. 10.1371/journal.pone.0015621 PMC3001486 21179448

[26] Schmidt I, Claesson CB, Westerholm B, Nilsson LG, Svarstad BL (1998). The impact of regular multidisciplinary team interventions on psychotropic prescribing in Swedish nursing homes. J Am Geriatr Soc.

[27] Crotty M, Halbert J, Rowett D, Giles L, Birks R, Williams H (2004). An outreach geriatric medication advisory service in residential aged care: a randomised controlled trial of case conferencing. Age Ageing.

[28] Opie J, Doyle C, O'Connor DW (2002). Challenging behaviours in nursing home residents with dementia: a randomized controlled trial of multidisciplinary interventions. Int J Geriatr Psychiatry.

[29] Davison TE, McCabe MP, Visser S, Hudgson C, Buchanan G, George K (2007). Controlled trial of dementia training with a peer support group for aged care staff. Int J Geriatr Psychiatry.

[30] Visser SM, McCabe MP, Hudgson C, Buchanan G, Davison TE, George K (2008). Managing behavioural symptoms of dementia: effectiveness of staff education and peer support. Aging Ment Health.

[31] Mitchell GK, Del Mar CB, O'Rourke PK, Clavarino AM (2008). Do case conferences between general practitioners and specialist palliative care services improve quality of life? A randomised controlled trial (ISRCTN 52269003). Palliat Med.

[32] Abernethy AP, Currow DC, Shelby-James T, Rowett D, May F, Samsa GP (2013). Delivery strategies to optimize resource utilization and performance status for patients with advanced life-limiting illness: results from the "palliative care trial" [ISRCTN 81117481]. J Pain Symptom Manage.

[33] Abernethy AP, Shelby-James T, Currow D, Williams H, Phillips P (2007). Individualised multi-disciplinary case conferences: can they be practically added to specialist palliative care? 8th Australian Palliative Care Conference; Sydney.

[34] Mitchell G, Cherry M, Kennedy R, Weeden K, Burridge L, Clavarino A (2005). General practitioner, specialist providers case conferences in palliative care--lessons learned from 56 case conferences. Aust Fam Physician.

[35] Mitchell GK, De Jong IC, Del Mar CB, Clavarino AM, Kennedy R (2002). General practitioner attitudes to case conferences: how can we increase participation and effectiveness?. Med J Aust.

[36] Halcomb EJ, Shepherd BM, Griffiths R (2009). Perceptions of multidisciplinary case conferencing in residential aged care facilities. Aust Health Rev.

[37] Holle D, Kruger C, Halek M, Sirsch E, Bartholomeyczik S. Experiences of nursing staff using dementia-specific case conferences in nursing homes. Am J Alzheimers Dis Other Demen. 2015;30(3):228–37. doi:http://dx.doi.org/10.1177/1533317514552320. 10.1177/1533317514552320 PMC10852842 25260597

[38] Craig P, Dieppe P, Macintyre S, Michie S, Nazareth I, Petticrew M et al. Developing and evaluating complex interventions: the new Medical Research Council guidance. BMJ. 2008;337:a1655. Also available open-source online at www.mrc.ac.uk/complexinterventionsguidance. doi:http://dx.doi.org/10.1136/bmj.a1655. 10.1136/bmj.a1655 PMC2769032 18824488

[39] Mid North Coast (NSW) Division of General Practice (MNCDGP), Coffs Harbour (2007). Toolkit: Creating a Multi-Disciplinary Team Approach to Care Planning In Residential Aged Care Facilities.

[40] Mid North Coast Division of General Practice (2006). Mid North Coast Rural Palliative Care Project: Final report.

[41] Campbell MK, Elbourne DR, Altman DG (2004). CONSORT statement: extension to cluster randomised trials. Bmj.

[42] Abernethy AP, Currow DC, Hunt R, Williams H, Roder-Allen G, Rowett D (2006). A pragmatic 2 x 2 x 2 factorial cluster randomized controlled trial of educational outreach visiting and case conferencing in palliative care-methodology of the Palliative Care Trial [ISRCTN 81117481]. Contemp Clin Trials.

[43] Roland M, Torgerson DJ (1998). What are pragmatic trials?. Bmj.

[44] Lurie JD, Morgan TS (2013). Pros and cons of pragmatic clinical trials. J Comp Effect Res.

[45] Australian Government Department of Social Services. myagedcare: Find a service. 2012. http://www.myagedcare.gov.au/service-finders#block-finder-aged-care-home-finder-agedcarehome. Accessed 12th November 2013.

[46] Reisberg B (1988). Functional assessment staging (FAST). Psychopharmacol Bull.

[47] Abernethy A, Shelby-James T, Fazekas B, Woods D, Currow D. The Australia-modified Karnofsky Performance Status (AKPS) scale: a revised scale for contemporary palliative care clinical practice. BMC Palliative Care. 2005;4(7):http://www.biomedcentral.com/content/pdf/1472-684X-4-7.pdf. 10.1186/1472-684X-4-7 PMC1308820 16283937

[48] Coventry PA, Grande GE, Richards DA, Todd CJ (2005). Prediction of appropriate timing of palliative care for older adults with non-malignant life-threatening disease: a systematic review. Age Ageing.

[49] Clay-Williams R, Nosrati H, Cunningham FC, Hillman K, Braithwaite J (2014). Do large-scale hospital- and system-wide interventions improve patient outcomes: a systematic review. BMC Health Serv Res..

[50] Ersek M, Kraybukk B, Hansen N (2006). Evaluation of a train-the-trainer program to enhance hospice and palliative care in nursing homes. J Hospice Palliat Nurs..

[51] Shaw EK, Howard J, West DR, Crabtree BF, Nease DE, Tutt B (2012). The role of the champion in primary care change efforts: from the State Networks of Colorado Ambulatory Practices and Partners (SNOCAP). J Am Board Fam Med.

[52] Wenger E, McDermott R, Snyder W (2002). Cultivating communities of practice: a guide to managing knowledge.

[53] Volicer L, Hurley AC, Blasi ZV (2001). Scales for evaluation of End-of-life care in dementia. Alzheimer Dis Assoc Disord.

[54] Kiely DK, Volicer L, Teno J, Jones RN, Prigerson HG, Mitchell SL (2006). The validity and reliability of scales for the evaluation of End-of-life care in advanced dementia. SO - Alzheimer Dis Assoc Disord.

[55] Parker D, Hodgkinson B (2010). A comparison of palliative care outcome measures used to assess the quality of palliative care provided in long-term care facilities: a systematic review. Pall Med.

[56] Mitchell SL, Kiely DK, Jones RN, Prigerson H, Volicer L, Teno JM (2006). Advanced dementia research in the nursing home: the CASCADE study. Alzheimer Dis Assoc Disord.

[57] Lindemann E (1994). Symptomatology and management of acute grief. 1944. Am J Psychiatry.

[58] van der Steen JT, Gijsberts MJ, Muller MT, Deliens L, Volicer L (2009). Evaluations of end of life with dementia by families in Dutch and U.S. nursing homes. Int Psychogeriatr.

[59] Weiner MF, Martin-Cook K, Svetlik DA, Saine K, Foster B, Fontaine CS (2000). The Quality of Life in Late-stage Dementia (QUALID) Scale. J Am Med Dir Assoc..

[60] Chenoweth L, King MT, Jeon Y-H, Brodaty H, Stein-Parbury J, Norman R (2009). Caring for Aged Dementia Care Resident Study (CADRES) of person-centred care, dementia-care mapping, and usual care in dementia: a cluster-randomised trial.[Erratum appears in Lancet Neurol. 2009 May;8(5):419]. Lancet neurol.

[61] Herdman M, Gudex C, Lloyd A, Janssen M, Kind P, Parkin D (2011). Development and preliminary testing of the new five-level version of EQ-5D (EQ-5D-5 L). Qual Life Res.

[62] Kind P, Spilker B (1996). The EuroQoL instrument: An index of health-related quality of life. Quality of life and pharmacoeconomics in clinical trials.

[63] Hounsome N, Orrell M, Edwards RT (2011). EQ-5D as a quality of life measure in people with dementia and their carers: evidence and key issues. Value in Health.

[64] Long CO, Sowell EJ, Hess RK, Alonzo TR. Development of the questionnaire on palliative care for advanced dementia (qPAD). Am J Alzheimers Dis Other Demen. 2012;27(7):537–43. doi:http://dx.doi.org/10.1177/1533317512459793. 10.1177/1533317512459793 PMC10697351 23002199

[65] Norman R, Cronin P, Viney R (2013). A pilot discrete choice experiment to explore preferences for EQ-5D-5 L health states. Appl Health Econ Health Pol.

[66] Norman GR, Sloan JA, Wyrwich KW (2003). Interpretation of changes in health-related quality of life: the remarkable universality of half a standard deviation. Med Care..

[67] Mitchell SL, Miller SC, Teno JM, Davis RB, Shaffer ML. The Advanced Dementia Prognostic Tool: A risk score to estimate survival in nursing home residents with advanced dementia. J Pain Symptom Manag. 2010;40(5):doi:10.1016/j.jpainsymman.2010.02.014. 10.1016/j.jpainsymman.2010.02.014 PMC2981683 20621437

[68] Ritchie J, Spencer L, Bryman A, Burgess RG (1994). Qualitative data analysis for applied policy research. Analyzing qualitative data.

[69] Strauss A, Corbin J (1998). Basics of Qualitative Research Techniques and Procedures for Developing Grounded Theory.

[70] Chenoweth L, Forbes I, Fleming R, King MT, Stein-Parbury J, Luscombe G (2014). PerCEN: a cluster randomized controlled trial of person-centered residential care and environment for people with dementia. Int Psychogeriatr.

[71] Chenoweth L, King MT, Jeon YH, Brodaty H, Stein-Parbury J, Norman R (2009). Caring for Aged Dementia Care Resident Study (CADRES) of person-centred care, dementia-care mapping, and usual care in dementia: a cluster-randomised trial. Lancet Neurol.

[72] Queensland University of Technology. ENABLE Project: Developing and testing a toolkit for the implementation and evaluation of person-centred evidence-based responses to need-driven behaviours associated with dementia. 2015. https://www.qut.edu.au/research/research-projects/enable-project-toolkit-dementia. Accessed 9th September 2015.

[73] Chenoweth L, King M, Luscombe G, Forbes I, Jeon YH, Parbury JS (2011). Study protocol of a randomised controlled group trial of client and care outcomes in the residential dementia care setting. Worldviews Evid Based Nurs.

[74] Chenoweth L, Jeon YH, Stein-Parbury J, Forbes I, Fleming R, Cook J et al. PerCEN trial participant perspectives on the implementation and outcomes of person-centered dementia care and environments. Int Psychogeriatr. 2015:1–13. doi:10.1017/s1041610215001350. 10.1017/S1041610215001350 26307245

[75] Aged Care Branch (2011). Dementia-friendly environments: residential care.

[76] Alzheimer’s Disease International (2013). World Alzheimer Report 2013. Journey of Caring: An analysis of long-term care for dementia.

[77] Chenoweth L, Kilstoff K (2002). Organizational and structural reform in aged care organizations: empowerment towards a change process. J Nurs Manag.

[78] Department of Health and Ageing. Aged Care Funding Instrument (ACFI). 2010. www.dss.gov.au/our-responsibilities/ageing-and-aged-care/tools-andresources/aged-care-funding-instrument-acfi-reports. Accessed Dec 12th 2010.

[79] Bellelli G, Frisoni GB, Bianchetti A, Trabucchi M (1997). The Bedford Alzheimer Nursing Severity scale for the severely demented: validation study. Alzheimer Dis Assoc Disord.

[80] Burke C (2012). Development of the person-centred environment and care assessment tool (PCECAT) and guidelines [PhD].

[81] Eagar K, Watters P, Currow DC, Aoun SM, Yates P (2010). The Australian Palliative Care Outcomes Collaboration (PCOC)--measuring the quality and outcomes of palliative care on a routine basis. Aust Health Rev.

